# Defects and lithium migration in Li_2_CuO_2_

**DOI:** 10.1038/s41598-018-25239-5

**Published:** 2018-04-30

**Authors:** Apostolos Kordatos, Navaratnarajah Kuganathan, Nikolaos Kelaidis, Poobalasuntharam Iyngaran, Alexander Chroneos

**Affiliations:** 1 Faculty of Engineering, Environment and Computing, Coventry University, Priory Street, Coventry, CV1 5FB United Kingdom; 2 Department of Materials, Imperial College London, London, SW7 2AZ United Kingdom; 3 Department of Chemistry, University of Jaffna, Sir. Pon Ramanathan Road, Thirunelvely, Jaffna, Sri Lanka

## Abstract

Li_2_CuO_2_ is an important candidate material as a cathode in lithium ion batteries. Atomistic simulation methods are used to investigate the defect processes, electronic structure and lithium migration mechanisms in Li_2_CuO_2_. Here we show that the lithium energy of migration via the vacancy mechanism is very low, at 0.11 eV. The high lithium Frenkel energy (1.88 eV/defect) prompted the consideration of defect engineering strategies in order to increase the concentration of lithium vacancies that act as vehicles for the vacancy mediated lithium self-diffusion in Li_2_CuO_2_. It is shown that aluminium doping will significantly reduce the energy required to form a lithium vacancy from 1.88 eV to 0.97 eV for every aluminium introduced, however, it will also increase the migration energy barrier of lithium in the vicinity of the aluminium dopant to 0.22 eV. Still, the introduction of aluminium is favourable compared to the lithium Frenkel process. Other trivalent dopants considered herein require significantly higher solution energies, whereas their impact on the migration energy barrier was more pronounced. When considering the electronic structure of defective Li_2_CuO_2_, the presence of aluminium dopants results in the introduction of electronic states into the energy band gap. Therefore, doping with aluminium is an effective doping strategy to increase the concentration of lithium vacancies, with a minimal impact on the kinetics.

## Introduction

The requirement for solid-state lithium batteries with better capacity, safety, cycle performance and durability have generated interest to materials with high lithium ion conductivity^1–12^. Oxide materials are actively being considered for energy applications and defect engineering is an effective way to improve their properties and performance^13,14^.

Li_2_CuO_2_ is an attractive material with many interesting characteristics, already considered for CO_2_ chemisorption^15,16^. In battery applications, a phase transition during the first circle of charge/discharge is confirmed and attributed to oxygen loss and delithiation. Recently, Ramos-Sanchez *et al*.^17^, have reported on this phase transition control and proposed Li_2_CuO_2_ for future applications due to its high capacity in pre-charge conditions as well as fast Li^+^ mobility. The partial pressure chemisorption has been also investigated by Lara-Garcia and Pfeiffer^18^. In essense, it was found that for low pressures the material’s behavior is not affected while increase of pressure combined with additional oxygen source enhances the CO_2_ chemisorption^18^. Furthermore, The Li^+^ and O^2-^ ion-diffusion can also affect the CO_2_ chemisorption by means of an extra layer that forms on the surface which limits the process as the CO_2_ cannot easily reach the bulk material.

In previous studies, the electronic structure of Li_2_CuO_2_ has been investigated using the local density approximation (LDA)^19^, LDA—LCAO^20^ and the generalized gradient approximation (GGA)^21^, however, there is lack of clarity in the literature for the exact contributions of each element (and their orbitals) in the valence and conduction band. Ramos-Sanchez *et al*.^17^ report on the band gap formation with higher oxygen states close to the Fermi level. To further examine the electronic structure and the defect processes of Li_2_CuO_2_, atomistic simulations are required. These can identify the defect engineering processes which are able to lead to the improvement of the material properties and accelerate progress^22–25^.

In the present study, we investigate the intrinsic defect processes, electronic structure and lithium vacancy self-diffusion in Li_2_CuO_2_ using static atomistic simulations and density functional theory (DFT).

## Results and Discussion

### Li_2_CuO_2_ structure

**Figure 1:**
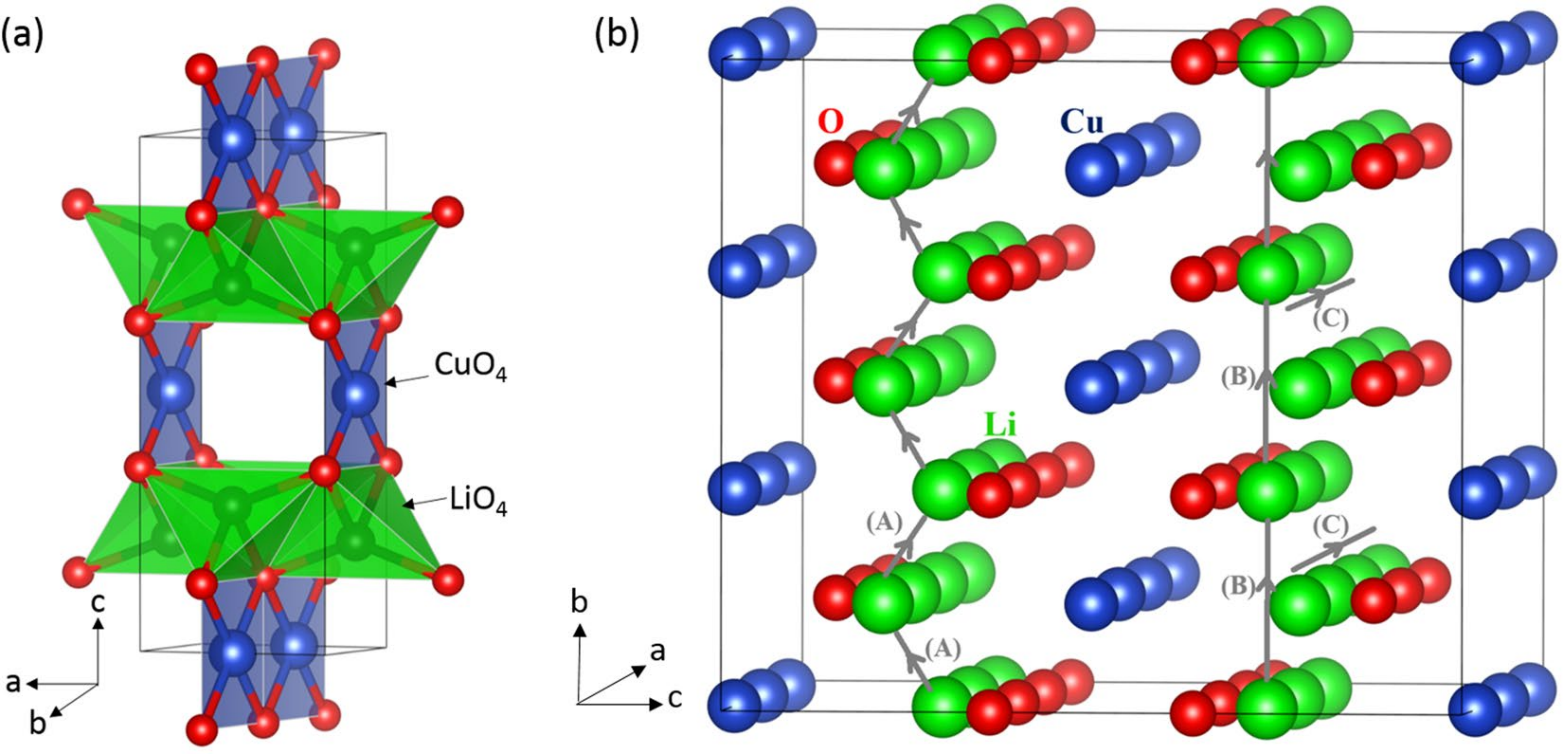
(**a**) Crystal structure of Li_2_CuO_2_ (space group *Immm*) and (**b**) the possible lithium ion migration paths considered. Li, Cu, and O atoms are shown as green, blue and red spheres respectively.

Figure 1(a) presents the crystallographic structure of Li_2_CuO_2_. It is characterized by the body centered orthorombic structure of the *Immm* space group (lattice parameters a = 3.6615 Å, b = 2.7887 Å and c = 9.5734 Å) as reported by Sapiña *et al*.^26^ Four O^−2^ atoms reside around each Cu^+2^ atom for the bc-plane while the square-shaped CuO_2_ present a shared edge on the b-axis. The interatomic potentials used in this study accurately reproduce the experimental lattice parameters, angles and bond lengths within an error margin less than 2.6% (refer to Tables S1 and S2 in the Supplementary Information).

### Intrinsic defect processes

The study of the intrinsic defect processes of energy materials is important in order to understand their electrochemical behavior and their propensity to form intrinsic defects. The intrinsic defect reactions in Kröger-Vink notation are:

1 $$Li\,Frenkel:L{i}_{Li}^{X}\to {V}_{Li}^{\text{'}}+\,L{i}_{i}^{\bullet }$$

2 $$O\,Frenkel:{O}_{O}^{X}\to {V}_{O}^{\bullet \bullet }+\,{O}_{i}^{\text{'}\text{'}}$$

3 $$Cu\,Frenkel:C{u}_{Cu}^{X}\to {V}_{Cu}^{\text{'}\text{'}}+C{u}_{i}^{\bullet \bullet }$$

4 $$Schottky:2\,L{i}_{Li\,}^{X}+C{u}_{Cu}^{X\,}+2\,{O}_{O}^{X}\to \,2\,{V}_{Li}^{\text{'}}+\,{V}_{Cu}^{\text{'}\text{'}}+2\,{V}_{O}^{\bullet \bullet }+L{i}_{2}Cu{O}_{2}$$

5 $$L{i}_{2}O\,Schottky:2\,L{i}_{Li}^{X}+\,{O}_{O}^{X\,}\to 2{V}_{Li}^{\text{'}}+{V}_{O}^{\bullet \bullet }+\,L{i}_{2}O$$

6 $$Li/Cu\,antisite\,(isolated):L{i}_{Li}^{X}+\,C{u}_{Cu}^{X\,}\to L{i}_{Cu}^{\text{'}}+C{u}_{Li}^{\bullet }$$

7 $$Li/Cu\,antisite\,(cluster):L{i}_{Li}^{X}+L{a}_{La}^{X}\to {\{L{i}_{Cu}^{\text{'}}:C{u}_{Li}^{\bullet }\}}^{X}$$

The reaction energies for these intrinsic defect processes are reported in Table 1. Typically to most oxides, the formation of all Frenkel and Schottky defects is unfavourable and this suggests that the formation of vacancies and interstitial defects will be hindered at equilibrium conditions. Therefore, these intrinsic defects will be present only at low concentrations in undoped Li_2_CuO_2_. The lithium Frenkel is a lower energy process compared to the other Frenkel and Schottky processes considered. The formation enthalpy of Li_2_O via the Li_2_O Schottky-like reaction (relation 5) is a processes that requires an energy of 2.57 eV per defect (refer to Table 1). This is a process that can lead to further $\,{V}_{Li}^{\text{'}}$ and ${V}_{O}^{\bullet \bullet }$ however at elevated temperatures far from room temperature. We also considered the formation of antisite defects ($L{i}_{Cu}^{\text{'}}\,{\rm{and}}\,C{u}_{Li}^{\bullet }$) both when the defects are apart (isolated form, relation 6) and when they form a defect pair (cluster, relation 7). For the isolated case, the defect energies of the two antisites were calculated independently in order to calculate the energy of the defect process applying relation 6 (i.e. the effect of defect association is not included), which is 0.88 eV per defect (refer to Table 1). For the antisite pair (relation 7) the energy per defect drops considerably (0.31 eV per defect, refer to Table 1) mainly due to the binding of the oppositively charged defects and relaxation effects.

**Table 1:** Energetics of intrinsic defects in Li_2_CuO_2_.

Defect Process	Equation	Defect energy (eV)	Defect energy (eV)/defect
Li Frenkel	1	3.76	1.88
O Frenkel	2	6.52	3.26
Cu Frenkel	3	8.86	4.43
Schottky	4	17.9	3.59
Li_2_O Schottky- like	5	7.72	2.57
Li/Cu anti-site (isolated)	6	1.76	0.88
Li/Cu anti-site (cluster)	7	0.62	0.31

### Lithium self-diffusion

**Figure 2:**
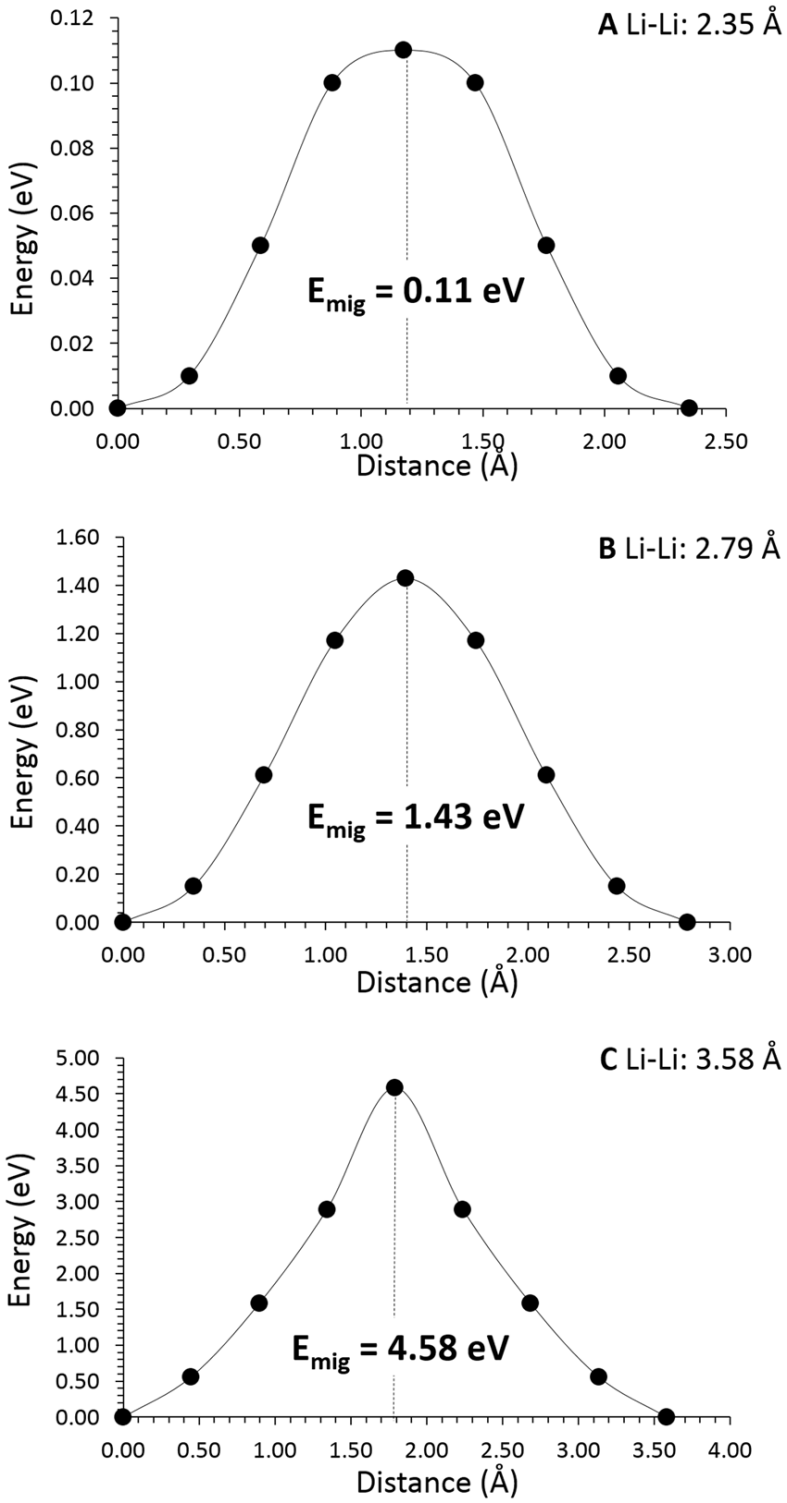
Three different energy profiles [as shown in Fig. 2 for Li diffusion paths (A, B and C)] of Li vacancy hopping between two adjacent Li sites in Li_2_CuO_2_. E_mig_ corresponds to the activation energy for the Li ion migration.

The present static atomistic simulation enabled the examination of possible Li vacancy migration paths and calculated the corresponding activation energies of migration. For the Li vacancy migration, we identified three paths between adjacent Li sites (refer to Fig. 1(b)). The zig-zag diffusion path (path A in Fig. 1(b)) in the ab-plane is the lowest energy process with an activation energy of migration of 0.11 eV (refer to Fig. 2). Here the activation energy of migration is defined as the position of the highest potential energy along the migration path.

### Trivalent doping

Although there is a very low migration activation energy the diffusion of lithium will be constrained by the high Li Frenkel energies, since there will only be a limited concentration of $\,{V}_{Li}^{\text{'}}$ that act as vehicles mediating Li self-diffusion via the vacancy mechanism. A defect engineering strategy to introduce a high $\,{V}_{Li}^{\text{'}}$ is by doping with trivalent ions. This can be described in the Kröger-Vink notation as:

8 $${R}_{2}{O}_{3}+2C{u}_{Cu}^{X}+2L{i}_{Li}^{X}\to \,2\,{R}_{Cu}^{\bullet }+2\,{V}_{Li}^{\text{'}}+\,2CuO+L{i}_{2}O\,$$

**Figure 3:**
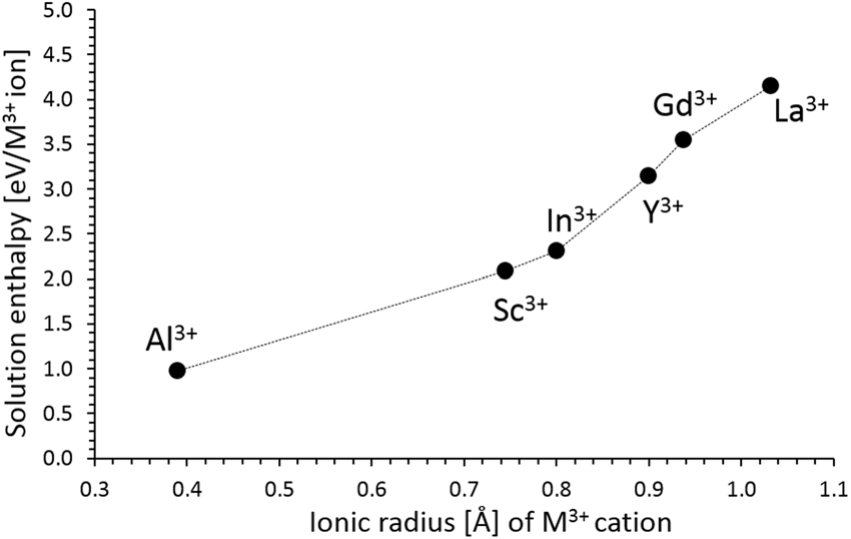
Enthalpy of solution of ${{R}}_{2}{{O}}_{3}$ (*R* = Al, Sc, In, Y, Gd and La) in Li_2_CuO_2_.

It should be noted that similar defect engineering strategy has been successfully employed in other energy materials such as materials for solid oxide fuel cells^27,28^. Here we have examined a number of ${R}_{2}{O}_{3}$ oxides, namely those with *R* = Al, Sc, In, Y, Gd and La, to find the oxide with the minimum solution enthalpy. The results of our calculations are shown in Fig. 3 where it can be observed that the solution energy of Al_2_O_3_ is the lowest one (0.97 eV/Al^3+^). Therefore, doping with Al will result in the formation of ${V}_{Li}^{\text{'}}\,\,\,$with energies significantly lower than those required by the Li Frenkel reaction. This will lead to a non-equilibrium concentration of ${V}_{Li}^{\text{'}}$. These vacancies will in turn act as vehicles for Li self-diffusion, increasing the Li diffusivity.

It is shown here that the Al_2_O_3_ solution enthalpy is considerably lower than the enthalpies for Schottky and Frenkel disorder. Therefore, vacancy and interstitial concentrations will be primarily due to the extrinsic impurity compensation processes. Similar processes have been previously considered in ceramic materials for energy applications (solid oxide fuel cells), such as Y_2_O_3_ or doped CeO_2_^28,29^. It should also be noted that the defect enthalpies are expected to be overestimated (although relative energies will be very reliable) as (a) we assume a fully ionic model (refer to Table S1 in the Supplementary Information) and (b) the calculations correspond to the dilute limit.

**Figure 4:**
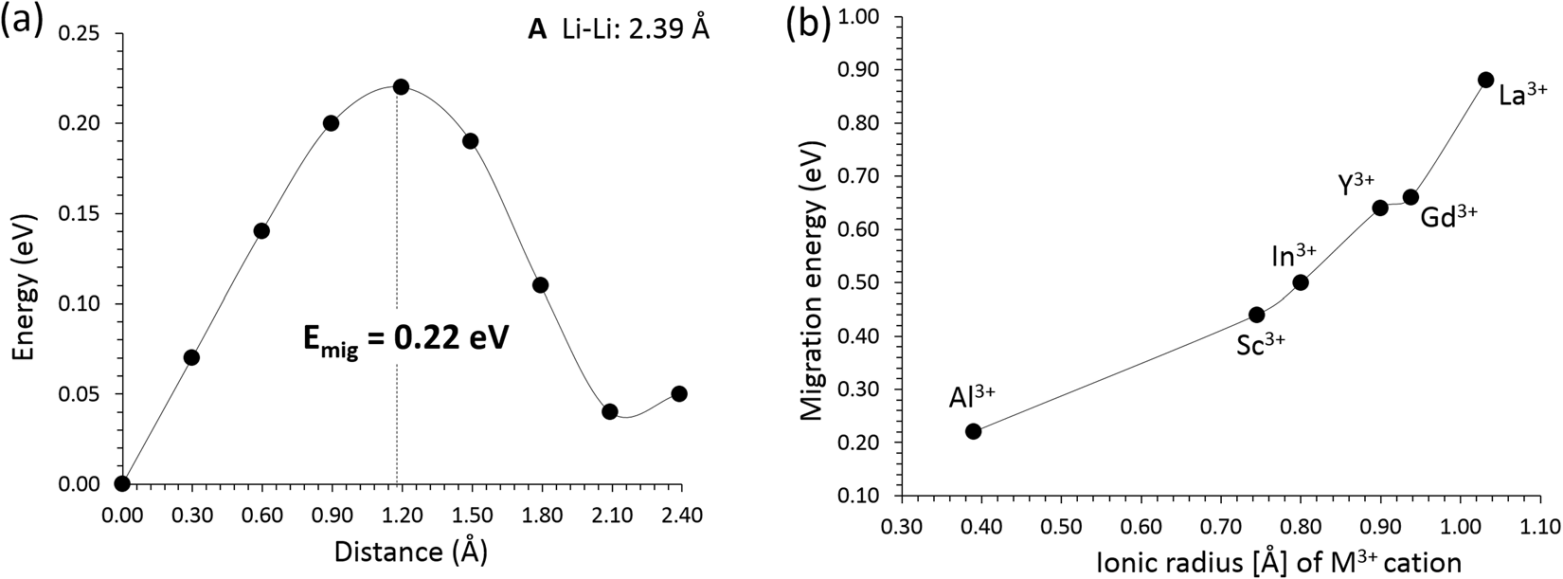
(**a**) The different energy profiles in the vicinity of an Al atom [effectively path (A) in Fig. 2 but with a nearest neighbour Al substitutional dopant] of Li vacancy hopping between two adjacent Li sites in Li_2_CuO_2_. (**b**) The activation energy of migration, E_mig_, of Li with respect to the ionic radius of the dopants (*R* = Al, Sc, In, Y, Gd and La) in the vicinity of the migrating Li ion.

The introduction of substitutional dopants in the lattice may also impact the migration energies of lithium. Figure 4(a) represents the different energy profiles in the vicinity of an Al dopant atom. It can be deduced that the introduction of dopants in the vicinity of the migrating ${V}_{Li}^{\text{'}}$ will increase the migration energy barriers from 0.22 eV (for the smallest dopant, Al) to 0.88 eV (for the largest dopant, La) (refer to Fig. 4 (b) and Figure S1 in the Supplementary Information). The largest increase of the migration energy barrier is for the largest dopant (i.e. La) and this is consistent with the larger association (i.e. relaxation of the large dopant in the vacant space) of the $\{L{a}_{Cu}^{\bullet }:{V}_{Li}^{\text{'}}$}^*X*^ pairs (−0.65 eV) as compared to the $\{A{l}_{Cu}^{\bullet }:{V}_{Li}^{\text{'}}$}^*X*^ pairs (−0.45 eV). This association in essense introduced an additional energetic barrier that needs to be overcome.

### Densities of states

**Figure 5:**
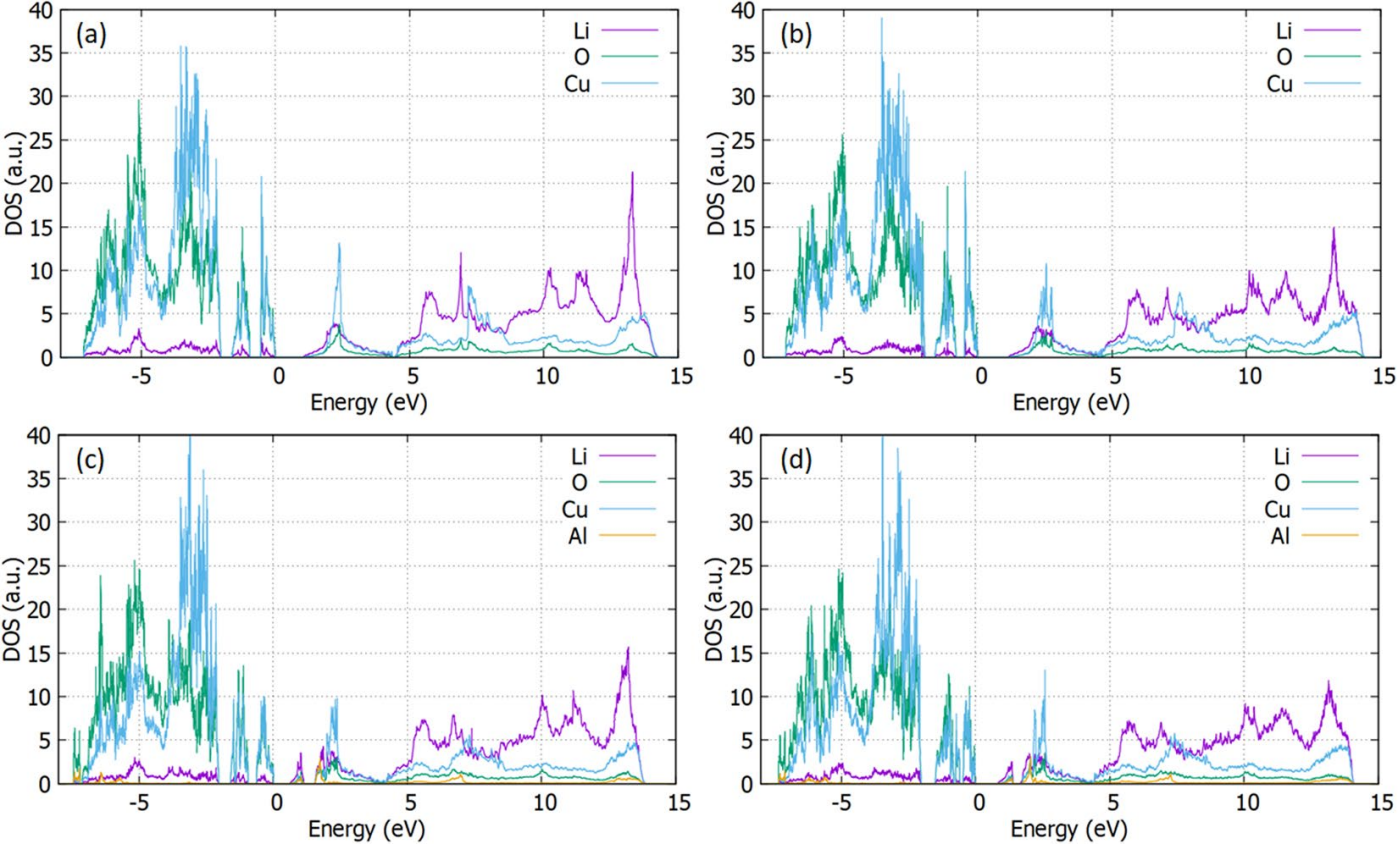
The densities of states for Li_2_CuO_2_: (**a**) perfect cell (**b**) ${{\boldsymbol{V}}}_{{\boldsymbol{Li}}}^{\text{'}}$ (**c**) ${\boldsymbol{A}}{{\boldsymbol{l}}}_{{\boldsymbol{Cu}}}^{\bullet }$ (**d**) ${\{{\boldsymbol{A}}{{\boldsymbol{l}}}_{{\boldsymbol{Cu}}}^{\bullet }:{{\boldsymbol{V}}}_{{\boldsymbol{Li}}}^{\text{'}}\}}^{X}$.

We have performed density of states (DOS) calculations and plotted the individual contribution of each element (partial-DOS) in Fig. 5, for the cases of (a) perfect cell (b) ${{\rm{V}}}_{{\rm{Li}}}^{\text{'}}$ (c) $A{l}_{Cu}^{\bullet }\,\,\,$and (d)$\,\{{{\rm{Al}}}_{{\rm{Cu}}}^{\bullet }:{{\rm{V}}}_{{\rm{Li}}}^{\text{'}}$}^X^. The valence band maximum (VBM) is set at zero energy level. For the perfect structure (refer to Fig. 5(a)), the band gap of Li_2_CuO_2_ is calculated at 1.05 eV. It is shown that Cu and O mainly contribute to the DOS of the valence band, driven by the Cu-d and O-p orbitals, while Li and Cu are the main contributors to the conduction band, driven by the Li-p and Cu-p orbitals (refer also to Figure S2 in the Supplementary Information). We also observe the high states of Cu and O just below the VBM and the non-uniformity of the band as presented by two separated contributions, in agreement with a previous study^17^. The effect on the DOS of the introduction of a lithium vacancy in the supercell is minimal (refer to Fig. 5(b)) as no additional states are created into the band gap, wheras only a small increase in the DOS in the conduction band due to Lithium is observed. (for oxygen vacancies refer to supplementary material Figure S3).

The impact of the $A{l}_{Cu}^{\bullet }\,\,\,$and $\{A{l}_{Cu}^{\bullet }:{V}_{Li}^{\text{'}}$}^*X*^ pairs on the densities of states on Li_2_CuO_2_ was also considered (refer to Fig. 5(c,d)). It is shown that the ${{\rm{Al}}}_{{\rm{Cu}}}^{\bullet }$ introduces a distribution of states in the band gap with a simultaneous minor narrowing of the gap itself (to 0.80 eV). Similar picture is observed in Fig. 5(d) for the $\{A{l}_{Cu}^{\bullet }:{V}_{Li}^{\text{'}}$}^*X*^ case (for ${R}_{Cu}^{\bullet }$ and $\{{R}_{Cu}^{\bullet }:{V}_{Li}^{\text{'}}$}^*X*^ please refer to Figures S4 and S5 in the Supplementary Information). Therefore, the introduction of Al (and other) dopants has an effect on the electronic structure, mainly with the introduction of states in the band gap but also with a minor narrowing of the gap. Although for battery applications, the leading conduction mechanism is the diffusion of ions, the effect of the energy levels in the band gap have to be considered as well.

## Summary

In the present study, the intrinsic defect processes and the Li vacancy diffusion in Li_2_CuO_2_ were investigated. Lithium vacancy diffusion with a very low migration energy (0.11 eV) along zig-zag diffusion path in the ab-plane is the lowest energy process. The high lithium Frenkel energy in essense implies a low concentration of lithium vacancies. We propose here doping Li_2_CuO_2_ with aluminium to introduce a higher concentration of lithium vacancies. The aluminium dopants will impact the activation energy of migration increasing the barrier to 0.22 eV. The combination of low solution enthalpy and low increase in the migration energy of lithium as compared to the intrinsic processes or the other dopants considered here, constitutes doping with aluminium the preferential doping strategy to increase the lithium diffusivity in Li_2_CuO_2_. The DOS analysis reveals that the introduction of aluminium will introduce electronic states into the energy band gap. It is anticipated that the present study will motivate further experimental and theoretical work^30–32^ to determine the diffusion properties of Li_2_CuO_2_ and its potential application in batteries. Additionally, it will generate further interest in lithium cuprate materials for energy storage applications.

## Methods

Atomistic simulations employing interatomic potentials method were used to investigate the relative energetics for the formation of intrinsic defects and the possible pathways for lithium ion migration. In particular Buckingham-type interatomic potentials as implemented in the general utility lattice program (GULP)^33^ were used, within the Born model of solids. The interactions between ions consist of a long-range Coulombic term and a short-range component, which aims to represent electron-electron repulsion and van der Waals interactions. Short-range interactions were modeled using the Buckingham potentials. The Broyden-Fletcher-Goldfarb-Shanno (BFGS) algorithm^34^ was employed to relax the simulation boxes and atomic positions. Lattice relaxation around point defects and the migrating ions were considered using the Mott-Littleton method^35^. This divides the system into two concentric regions, where the ions within the inner spherical region (>700 ions) surrounding the defect relaxed explicitly. The defect calculations were performed in supercells containing 720 ions.

Li diffusion was investigated by considering two adjacent vacancy sites as initial and final configurations. The Li interstitial ion was placed along the direct pathway between the initial and final vacancy configuration. We have considered seven intermediate (interstitial) positions which were fixed while all other ions were free to relax. The energy difference between the saddle point position and the system in its initial state is effectively the activation energy of migration.

To calculate the DOSs for pure and defective Li_2_CuO_2_, density functional theory simulations were employed using the plane wave code CASTEP^36,37^. The exchange and correlation interactions are modelled by using the corrected density functional of Perdew, Burke and Ernzerhof (PBE)^38^ in the generalized gradient approximation (GGA), with ultrasoft pseudopotentials^39^. The kinetic energy cut-off of the plane wave basis is 500 eV, in conjunction with a 3 × 2 × 3 Monkhorst-Pack (MP)^40^ *k*-point grid and a 60-atomic site supercell. To consider correlation effects of localized electrons onsite Coulomb repulsions in the range of 4–8 eV is set for the Cu 3d orbitals. We have tested this value to establish that the trends are not affected by the specific choice of U-parameter. The calculations were under constant pressure conditions. The system has been treated as spin polarized. For the PDOS analysis/imaging, the OPTADOS code is employed^41,42^.

## Electronic supplementary material

Supplementary Information
