# Supplementary material for: Defects and lithium migration in Li2CuO2
Source: Sci Rep. 2018 Apr 30;8:6754. doi: 10.1038/s41598-018-25239-5 (PMC5928103; doi:10.1038/s41598-018-25239-5)
Supplement: Supplementary file 1 — Supplementary Information [file 41598_2018_25239_MOESM1_ESM.docx]

**Supporting Information**

**Defects and lithium migration in Li_2_CuO_2_**

# Apostolos Kordatos,^1^ Navaratnarajah Kuganathan,^2,a)^^[[1]](#footnote-2)^ Nikolaos Kelaidis,^1^ Poobalasuntharam Iyngaran,^3^ & Alexander Chroneos,^1,2,b)*^

*^1^Faculty of Engineering, Environment and Computing, Coventry University, Priory Street, Coventry CV1 5FB, United Kingdom*

*^2^Department of Materials, Imperial College London, London SW7 2AZ, United Kingdom*

*^3^ Department of Chemistry, University of Jaffna, Sir. Pon Ramanathan Road, Thirunelvely, Jaffna, Srilanka*

**Table S1**. Interatomic potential parameters used in the atomistic simulations of Li_2_CuO_2_ and the dopants considered

Two-body [Φ*_ij_* (*r_ij_*) = *A_ij_* exp (− *r_ij_* /*ρ_ij_*) − *C_ij_ / r_ij_*^6^]

| Interaction | *A* (eV) | *ρ* (Å) | *C* (eV·Å^6^) | Y (e) | K (eV·Å^-2^) |
| --- | --- | --- | --- | --- | --- |
| Li^+^ - O^2−^ | 632.1018 | 0.2906 | 0.00 | 1.000 | 99999 |
| Cu^2+^ - O^2−^ | 3860.60 | 0.2427 | 0.00 | 2.000 | 99999 |
| O^2−^ - O^2−^ | 22764.30 | 0.1490 | 27.627 | –2.75823 | 30.211 |
| Al^3+^ - O^2−^ | 1725.20 | 0.28971 | 0.000 | 3.000 | 99999 |
| Sc^3+^ - O^2−^ | 1575.85 | 0.3211 | 0.000 | 3.000 | 99999 |
| In^3+^ - O^2−^ | 1495.65 | 0.3327 | 4.33 | 3.000 | 99999 |
| Y^3+^ - O^2−^ | 1766.40 | 0.33849 | 19.43 | 3.000 | 99999 |
| Gd^3+^ - O^2−^ | 1885.75 | 0.3399 | 20.34 | 3.000 | 99999 |
| La^3+^ - O^2−^ | 2088.79 | 0.3460 | 23.25 | 3.000 | 99999 |

**Table S2.** Calculated and Experimental (Sapiña, F. *et al.* Crystal and magnetic structure of Li_2_CuO_2_. *Solid State Commun.* **74**, 779-784 (1990)) Structural Parameters and Bond Distances for Orthorhombic (*Immm*) Li_2_CuO_2_

| Parameter | Calc | Expt | \|∆\|(%) |
| --- | --- | --- | --- |
| a (Å) | 3.5779 | 3.6615 | 2.28 |
| b (Å) | 2.8628 | 2.7887 | 2.59 |
| c (Å) | 9.3926 | 9.5734 | 1.92 |
| α = β = γ (°) | 90.00 | 90.00 | 0.00 |
| Li – O (Å) | 1.9560 | 1.9610 | 0.25 |
| Cu – O (Å) | 1.9162 | 1.9577 | 2.12 |

**Table S3.** Calculated cluster binding energies.

| Trivalent cation R in $R_{Cu}^{\bullet}$ + $V_{Li}^{'}$ → $\{R_{Cu}^{\bullet}:V_{Li}^{'}$}*^X^* | Cluster binding energies (eV)/defect |
| --- | --- |
| Al^3+^ | –0.45 |
| Sc^3+^ | –0.52 |
| In^3+^ | –0.54 |
| Y^3+^ | –0.59 |
| Gd^3+^ | –0.60 |
| La^3+^ | –0.65 |

**
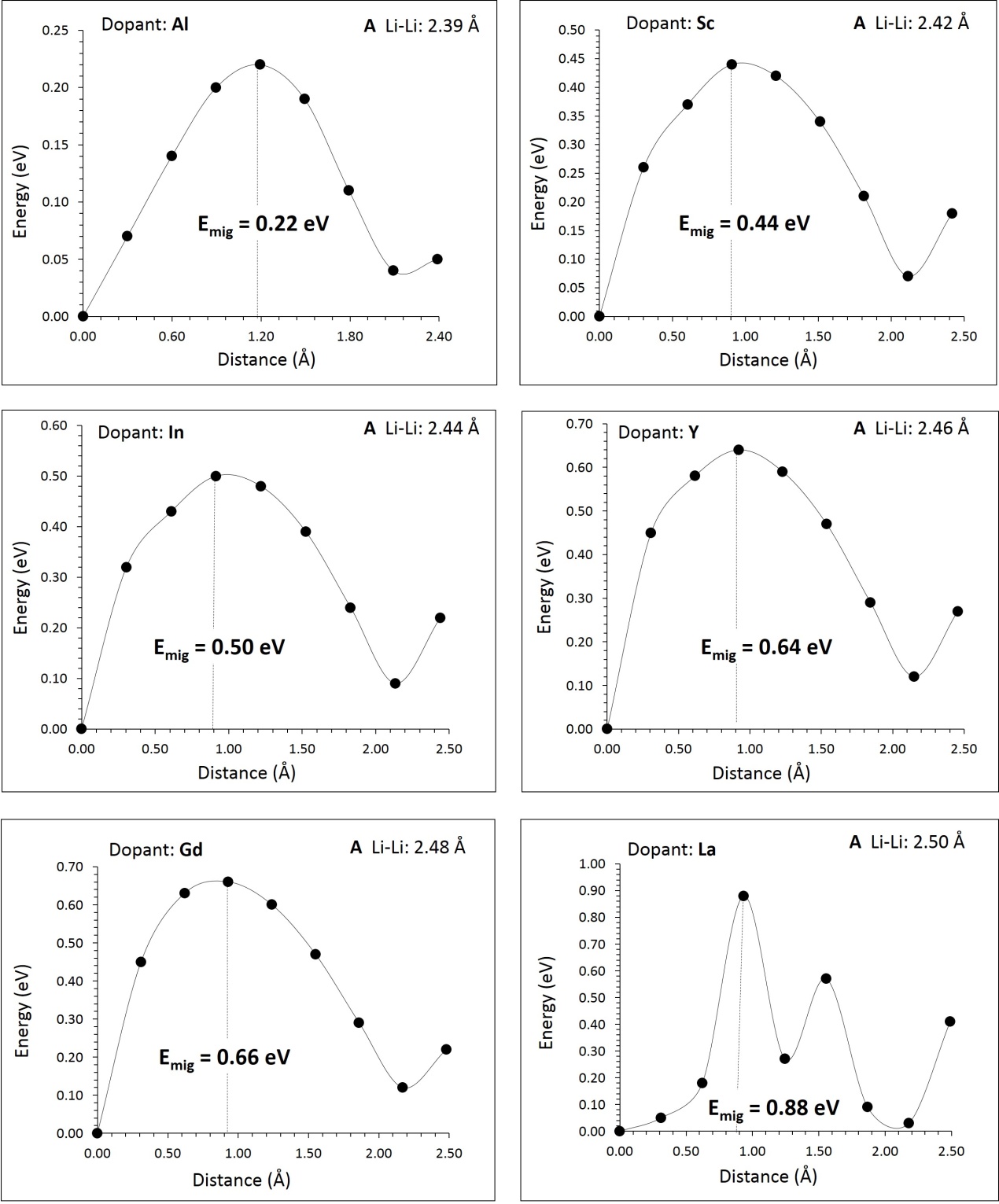
**

**Figure S1.** The different energy profiles in the vicinity of a dopant atom [effectively path (A) in Figure 2 but with a nearest neighbour substitutional dopant] of Li vacancy hopping between two adjacent Li sites in Li_2_CuO_2_.

**
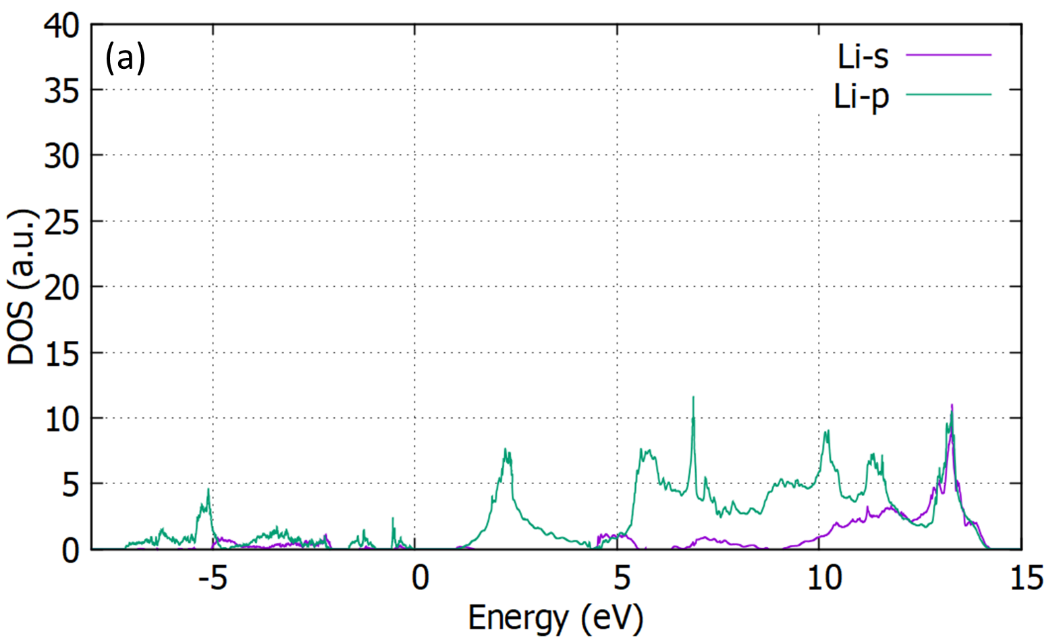
**

**
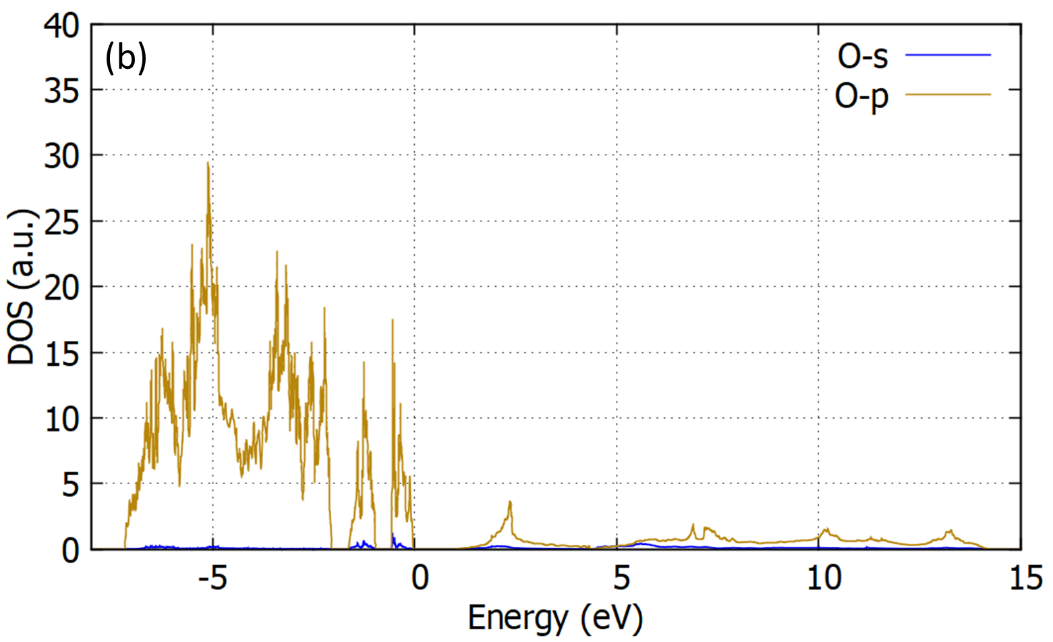
**

**
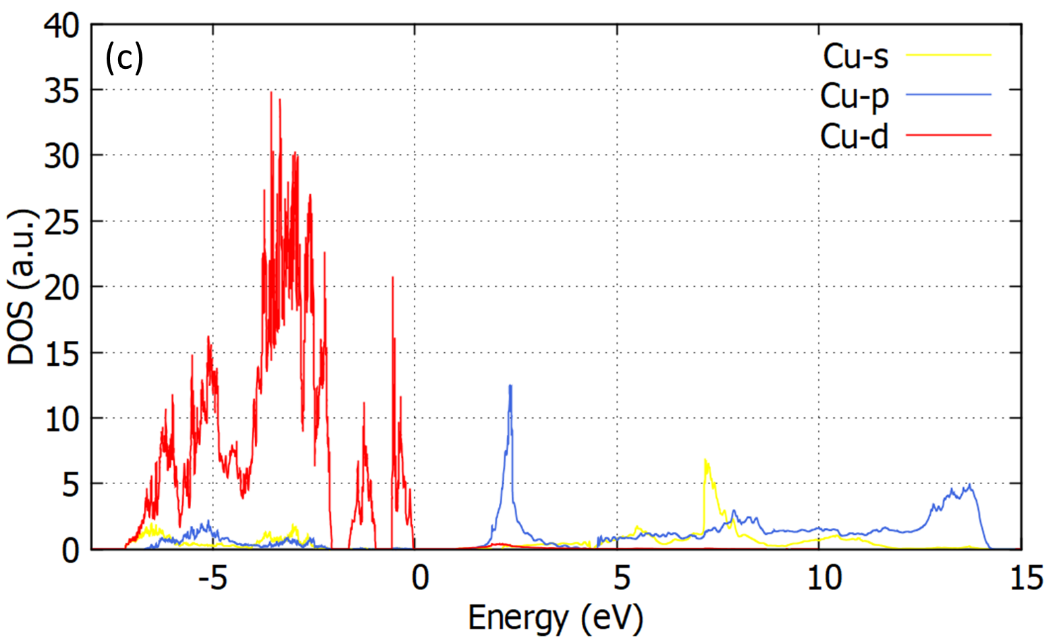
**

**Figure S2.** The contribution of the atomic orbitals to the partial densities of states of the perfect Li_2_CuO_2_ supercell shown in Figure 5(a).

**
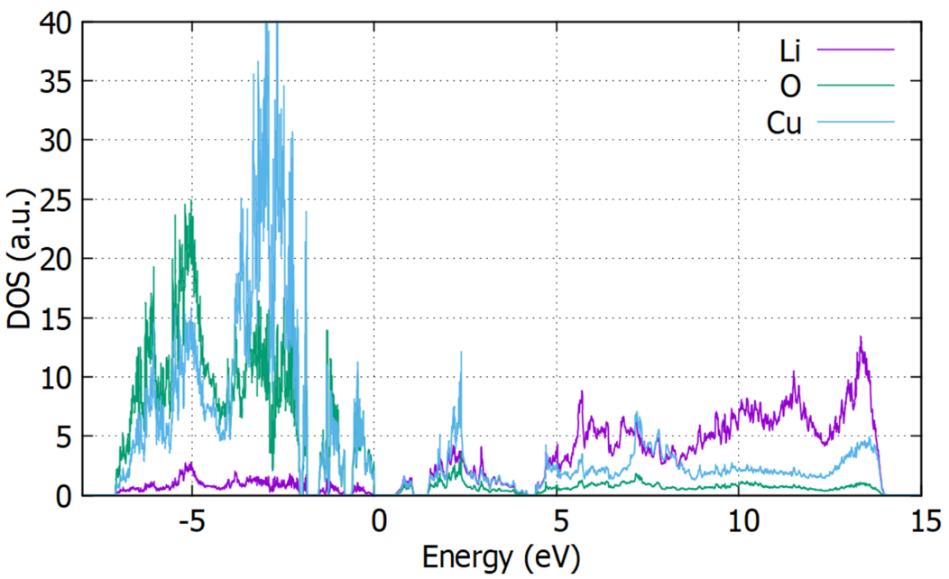
**

**Figure S3.** The densities of states in Li_2_CuO_2_ supercell when introducing oxygen vacancies.


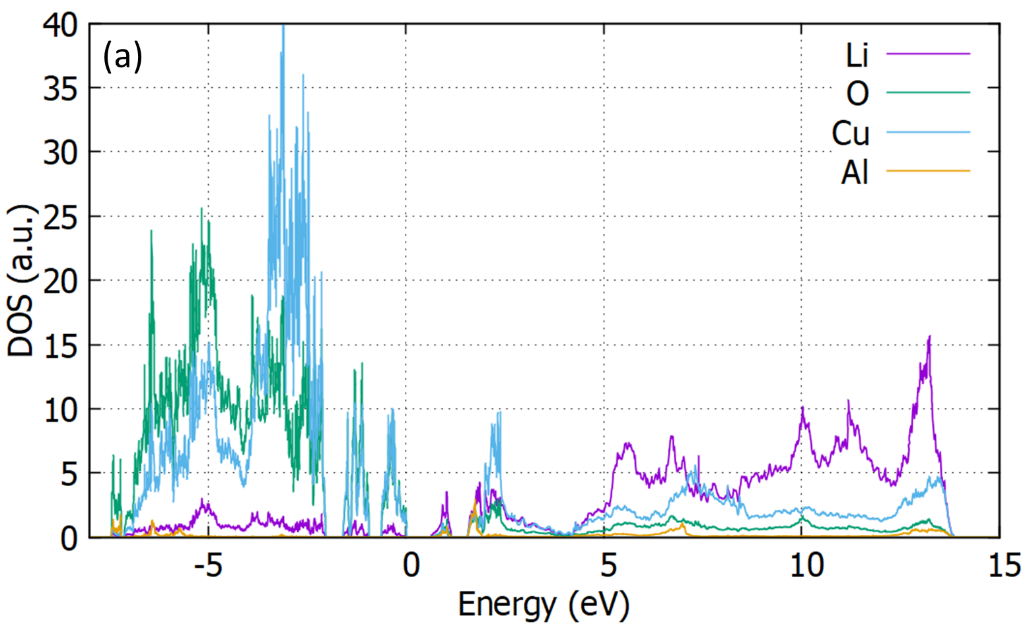


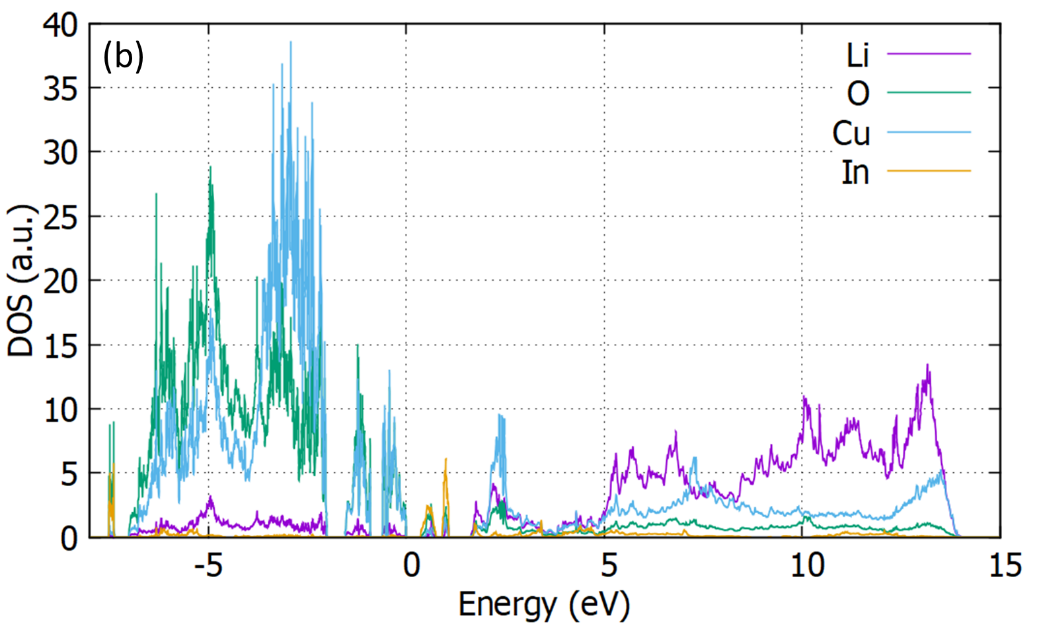


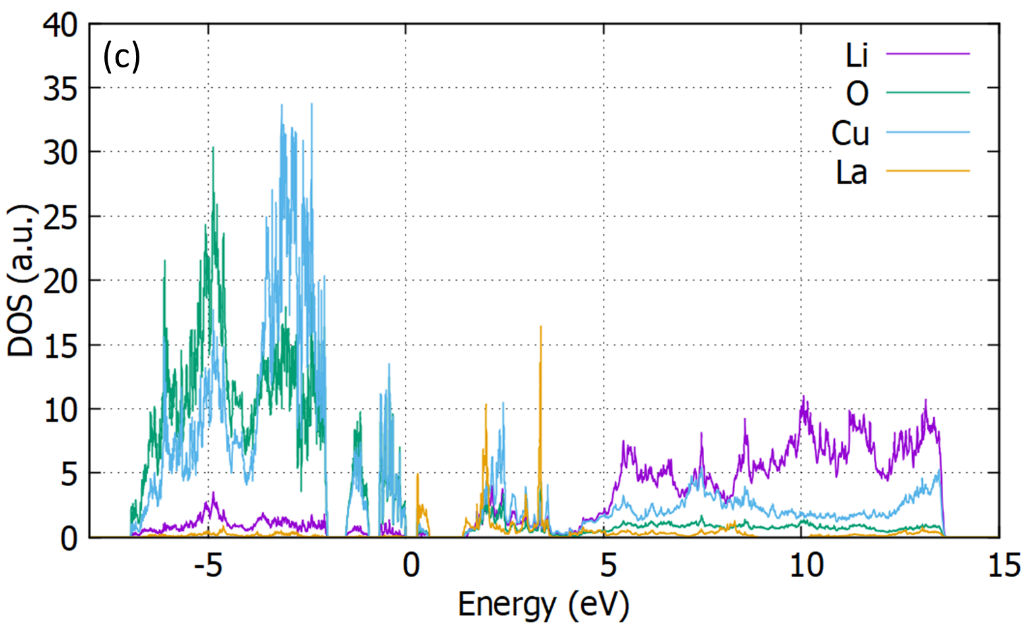


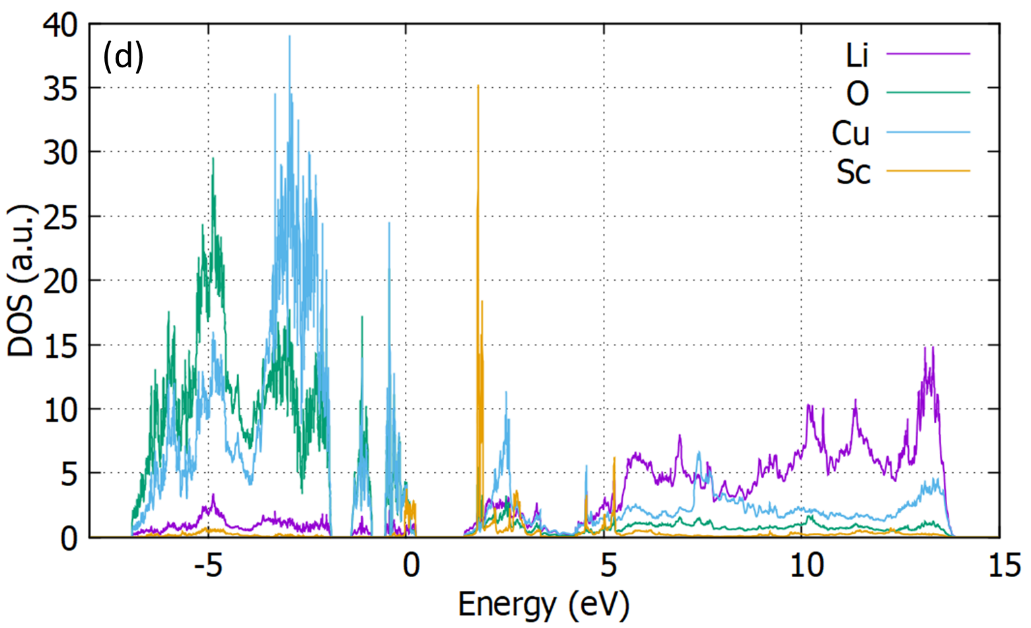


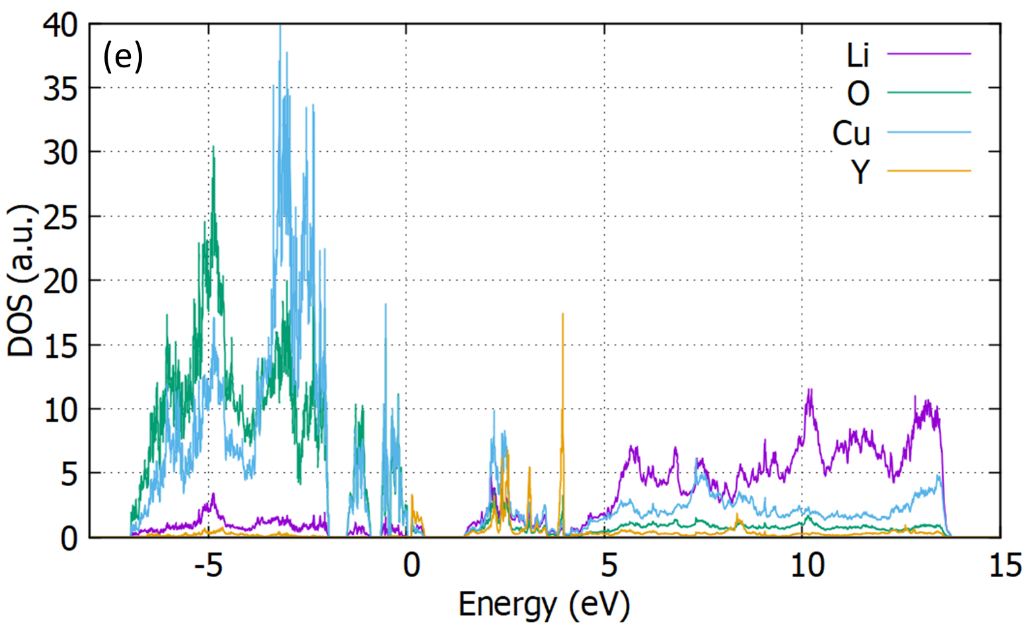


**Figure S4.** The densities of states for $R_{Cu}^{\bullet}$ in Li_2_CuO_2_ when R is (a) Al (b) In (c) La (d) Sc (e) Y.

**
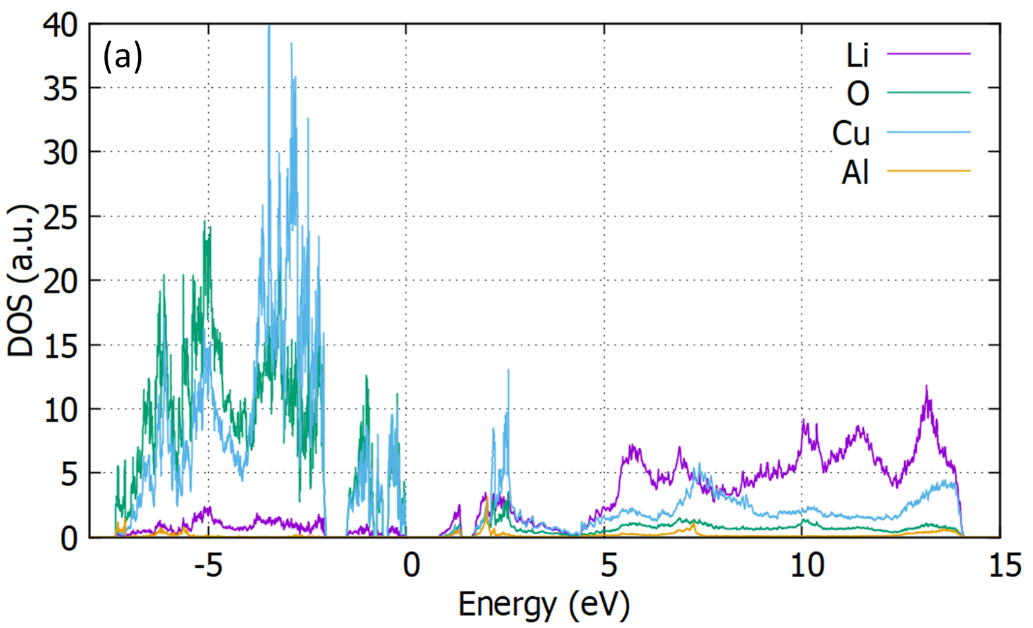
**

**
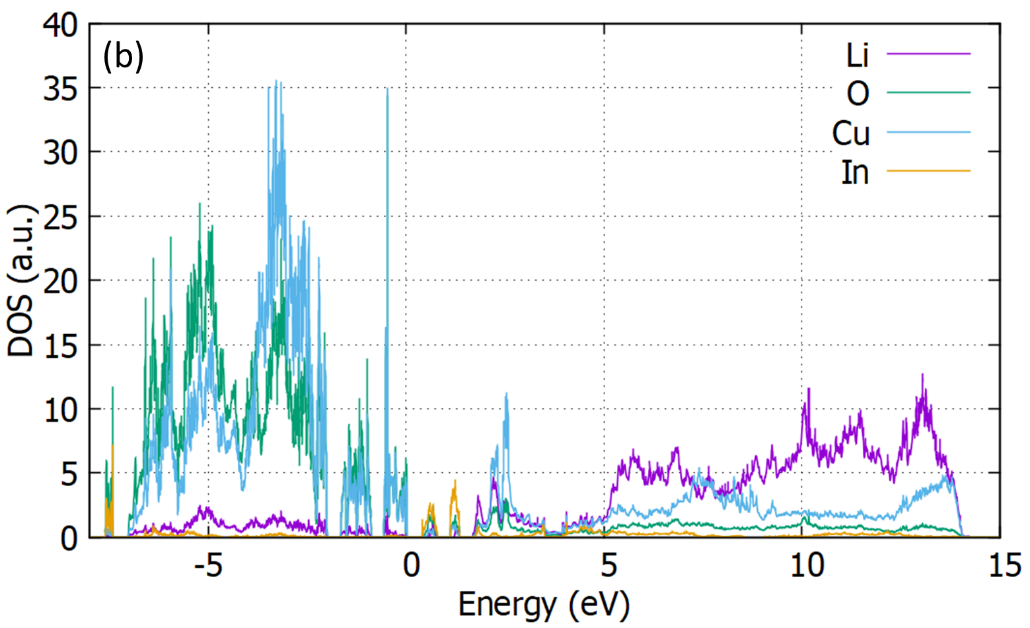
**

**
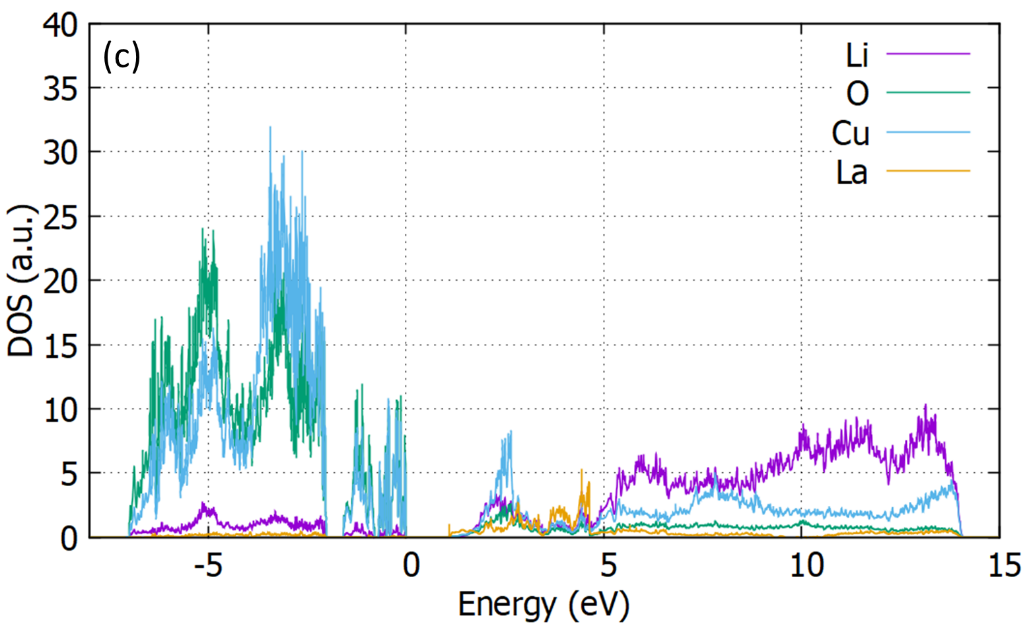
**

**
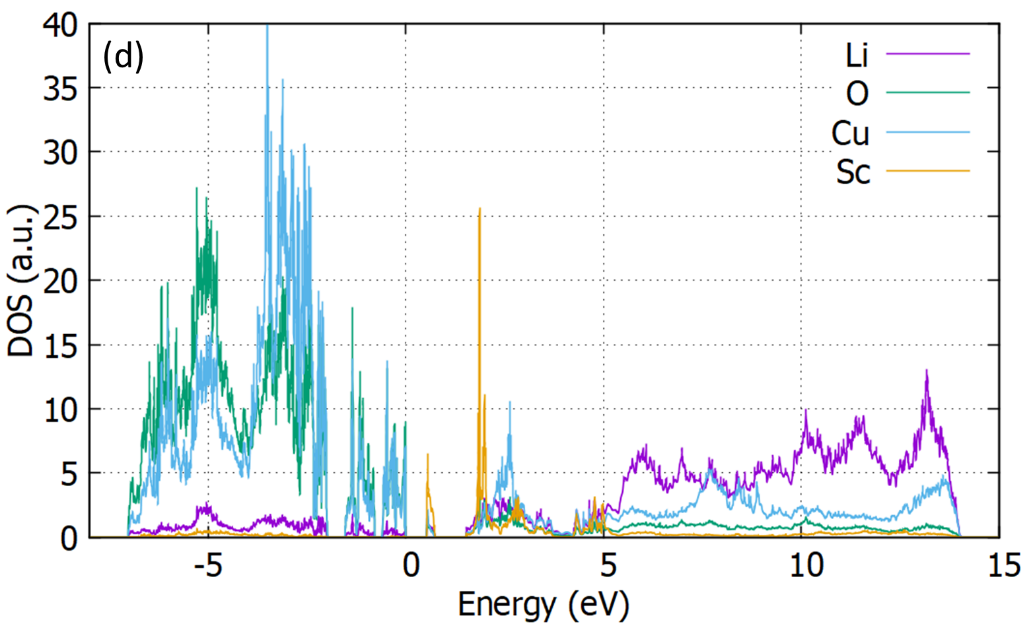
**

**
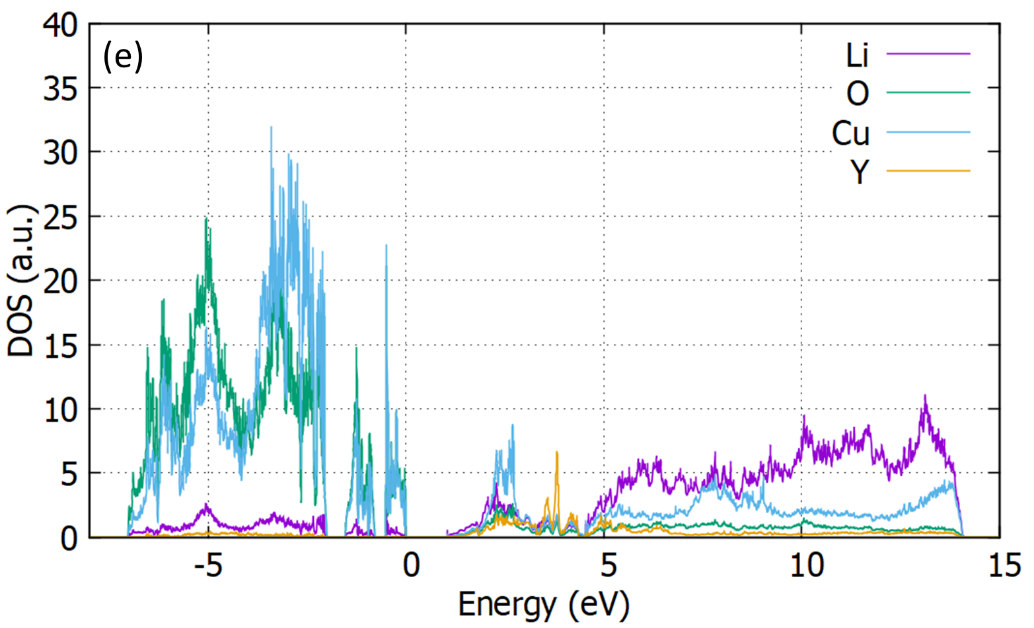
**

**Figure S5.** The densities of states for $\{R_{Cu}^{\bullet}:V_{Li}^{'}$}*^X^* in Li_2_CuO_2_ when R is (a) Al (b) In (c) La (d) Sc (e) Y.

1. Corresponding authors, e-mails: a) n.kuganathan@imperial.ac.uk

   b) [alexander.chroneos@imperial.ac.uk](mailto:alexander.chroneos@imperial.ac.uk) [↑](#footnote-ref-2)
